# Molecular Characterization of Three Novel Phospholipase A_2_ Proteins from the Venom of *Atheris chlorechis*, *Atheris nitschei* and *Atheris squamigera*

**DOI:** 10.3390/toxins8060168

**Published:** 2016-06-01

**Authors:** He Wang, Xiaole Chen, Mei Zhou, Lei Wang, Tianbao Chen, Chris Shaw

**Affiliations:** 1 School of Integrative Medicine, Fujian University of Traditional Chinese Medicine, No.1 Qiu Yang Road, Shangjie Town, Fuzhou 350122, Fujian, China; 2 School of Pharmacy, Fujian Medical University, No.1 Xueyuan Road, Shangjie Town, Fuzhou 350004, Fujian, China; 3 Natural Drug Discovery Group, School of Pharmacy, Queen’s University Belfast, University Road, Belfast BT7 1NN, UK; m.zhou@qub.ac.uk (M.Z.); l.wang@qub.ac.uk (L.W.); t.chen@qub.ac.uk (T.C.); chris.shaw@qub.ac.uk (C.S.)

**Keywords:** snake venom, phospholipase A_2_, molecular cloning, *Atheris*, mass spectrometry

## Abstract

Secretory phospholipase A_2_ (sPLA_2_) is known as a major component of snake venoms and displays higher-order catalytic hydrolysis functions as well as a wide range of pathological effects. *Atheris* is not a notoriously dangerous genus of snakes although there are some reports of fatal cases after envenomation due to the effects of coagulation disturbances and hemorrhaging. Molecular characterization of *Atheris* venom enzymes is incomplete and there are only a few reports in the literature. Here, we report, for the first time, the cloning and characterization of three novel cDNAs encoding phospholipase A_2_ precursors (one each) from the venoms of the Western bush viper (*Atheris chlorechis*), the Great Lakes bush viper (*Atheris nitschei*) and the Variable bush viper (*Atheris squamigera*), using a “shotgun cloning” strategy. Open-reading frames of respective cloned cDNAs contained putative 16 residue signal peptides and mature proteins composed of 121 to 123 amino acid residues. Alignment of mature protein sequences revealed high degrees of structural conservation and identity with Group II venom PLA_2_ proteins from other taxa within the Viperidae. Reverse-phase High Performance Liquid Chromatography (HPLC) profiles of these three snake venoms were obtained separately and chromatographic fractions were assessed for phospholipase activity using an egg yolk suspension assay. The molecular masses of mature proteins were all identified as approximately 14 kDa. Mass spectrometric analyses of the fractionated oligopeptides arising from tryptic digestion of intact venom proteins, was performed for further structural characterization.

## 1. Introduction

As major components of snake venoms, proteins of the secretory phospholipase A_2_ (sPLA_2_) family have been structurally-defined, characterized and their catalytic mechanisms have been elucidated [1]. Traditionally, these snake venom enzymes have been divided into two main groups, Group I (GI) and Group II (GII), used to distinguish between molecules based on their amino acid sequences, disulfide-bridging patterns, unique functional loops and extension amino acids [2]. Phospholipase A_2_ catalyzes the hydrolysis of the sn-2 fatty acyl bond of phospholipids in a calcium-dependent manner [3]. A broad range of physiological molecules, such as long-chain fatty acid phospholipids, short-chain fatty acid-containing oxidized phospholipids and platelet-activating factors, act as the natural substrates of phospholipase A_2_ [4]. The higher-order catalytic hydrolysis functions of these snake enzymes play a central role in lipid metabolism of numerous cells and tissues, contributing to prey digestion. In addition, much is known about the wide variety of pathological effects exhibited by the snake venom phospholipase A_2_s including their neurotoxicity, myotoxicity, cytotoxicity, anticoagulant effects, cardiotoxicity, hypotension induction, platelet aggregation/inhibition and anti-bacterial activities [5,6,7,8,9,10], which in many cases, are fundamental to the toxicity of snake venoms [11,12].

Of the 400 species of snakes in Africa, bush vipers (*Atheris*, Viperidae) are not considered to be highly-dangerous in terms of the numbers of documented bites and deaths. This may in part be due to their arboreal behavior and their distribution in often inhospitable habitats [13]. A full envenomation by *Atheris* is likely to be fatal, however, due to the injection of a large quantity of toxic venom through their long and sharp fangs [14]. Envenomations involving *Atheris* vipers are clinically-characterized by coagulation disturbances and hemorrhage, local pain, edema, bleeding and a prolongation of coagulation time and non-clotting blood [15,16]. Some victims develop acute renal failure and hypertension [17,18]. However, studies on characterization of venom enzymes to explain these sequelae, are scarce and are incomplete [19]. In fact, even superficial reports on the components of whole venoms are equally rare. Thus, hunting for and describing the toxic polypeptides and/or proteins in *Atheris* venoms became a focus of our interest. Novel disintegrins had been isolated and identified in a previous study [20], and in this work, cloning of precursor-encoding cDNAs and characterization of the mature phospholipase A_2_ proteins from *Atheris chlorechis* (*A. chlorechis*), *Atheris nitschei* (*A. nitschei*) and *Atheris squamigera* (*A. squamigera*) venoms, were carried out in an attempt to explain the toxic effects of the venom of *Atheris* snakes in more depth.

## 2. Results

### 2.1. cDNAs Encoding PLA_2_ Precursors and Bioinformatic Analyses

**Figure 1:**
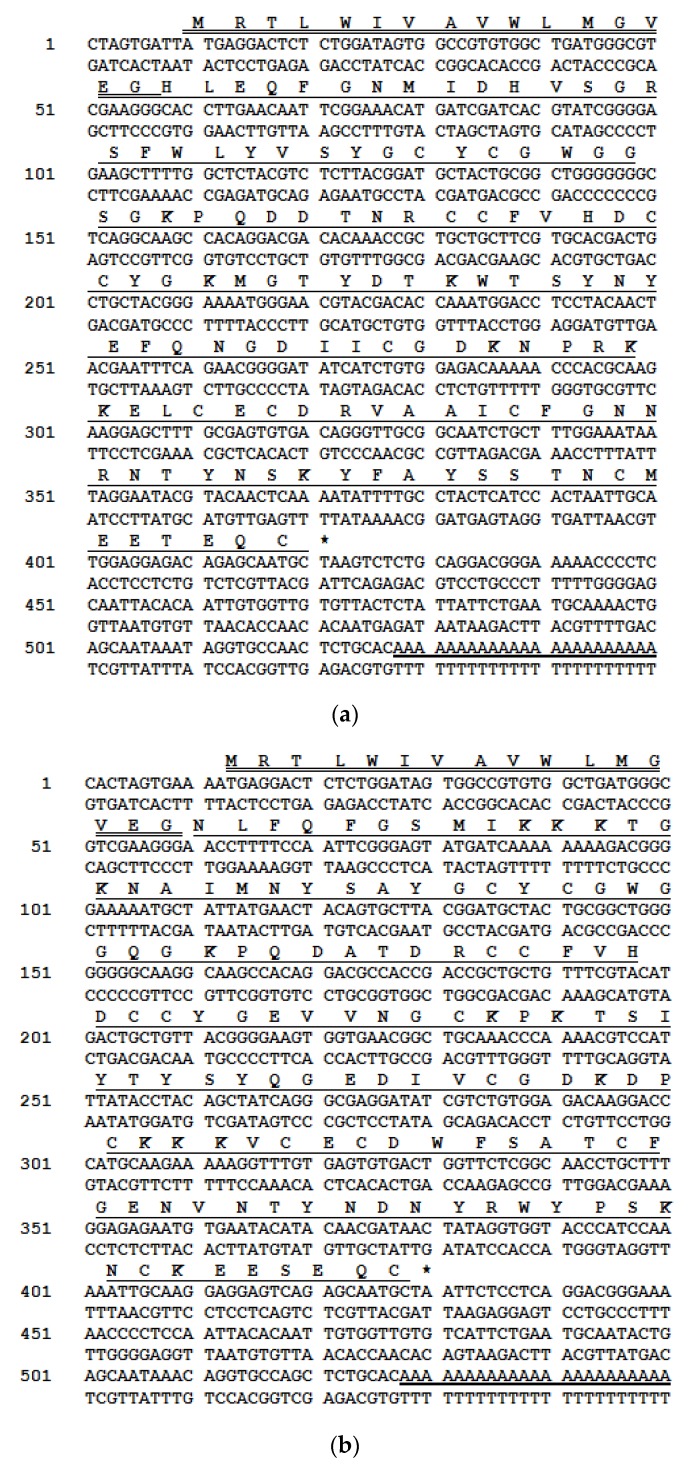

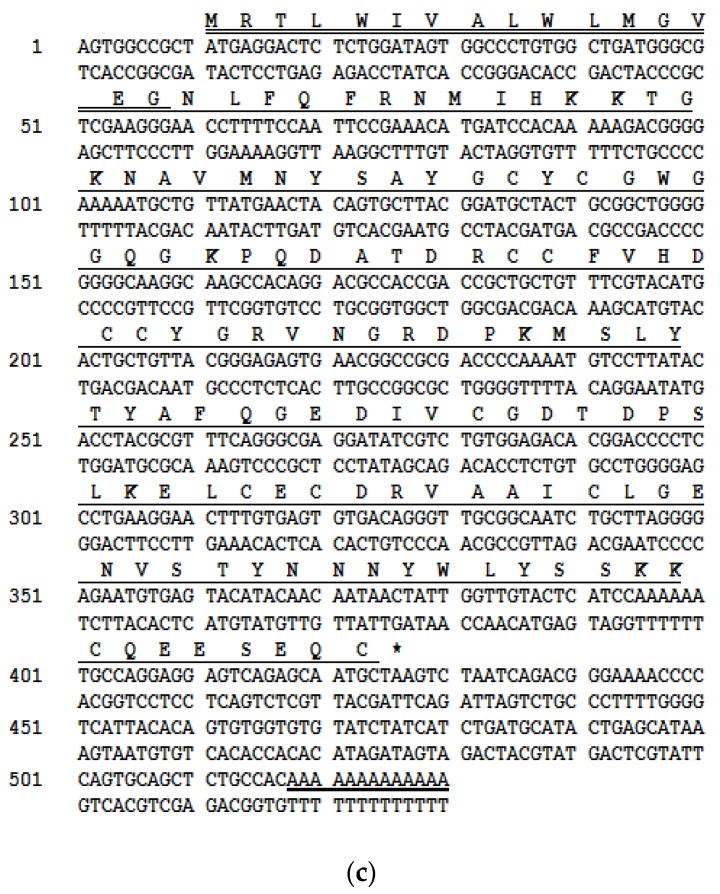
Nucleotide and translated amino acid sequences of the three Phospholipase A_2_ (PLA_2_) precursors: (**a**) PLA_2_-A.C. from *A. chlorechis*; (**b**) PLA_2_-A.N. from *A. nitschei*; and (**c**) PLA_2_-A.S. from *A. squamigera*. The putative signal sequences are double-underlined, the mature proteins are single-underlined and stop codons are indicated by asterisks. Polyadenylation sites are underlined.

A single novel unique full-length phospholipase-encoding precursor was cloned from each of the venom-derived libraries of *A. chlorechis*, *A. nitschei* and *A. squamigera* and each encoded a single copy of PLA_2_ (Figure 1). Here, the first genomic sequences for the three cDNAs encoding phospholipase A_2_ precursors from *Atheris* bush viper venoms, are reported and were named PLA_2_-A.C., PLA_2_-A.N. and PLA_2_-A.S., respectively. These data have been deposited in the Genbank Nucleotide Sequence Database under the accession codes, KP11968, KP119683 and KP119684, respectively. The open-reading frame amino acid sequences of these proteins exhibited a similar structural pattern: all contained a putative 16-residue signal peptide sequence and a mature protein sequence consisting of 123 residues for *A. nitschei*, 121 residues for *A. chlorechis* and 122 residues for *A. squamigera*. All contained a conserved Histidine/Aspartic acid dyad-motif and a cysteine-rich sequence that defines the PLA_2_ hydrolytic function.

**Figure 2:**
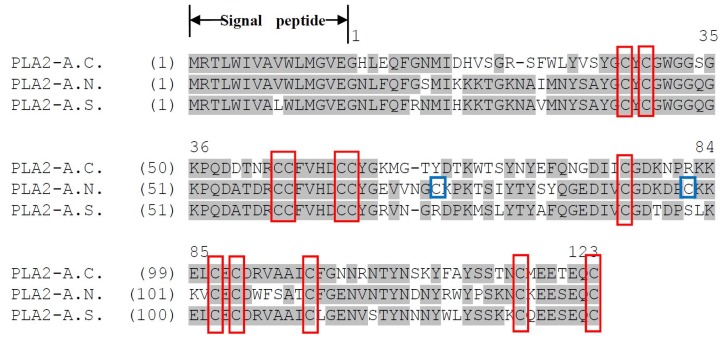
Alignment of phospholipase A_2_ proteins from the venoms of *A. chlorechis*, PLA_2_-A.C.; *A. nitschei*, PLA_2_-A.N.; and *A. squamigera*, PLA_2_-A.S. Identical amino acid residues are shaded gray. Putative signal peptides are marked and positionally-conserved cysteines in all of the proteins are outlined in red boxes, with the two additional cysteine residues outlined by blue boxes. The numbering of amino acid residues is indicated above the first sequence, and gaps are shown as dashes.

Comparison of these three precursors, established by deduction from cloned cDNA, is shown in the alignment in Figure 2. They exhibit a remarkable degree of identity: the signal peptides differ by only one residue, with the mature proteins showing high similarity especially in the features of the catalytic network and the primary metal binding site. Except for PLA_2_-A.N., with two additional cysteine residues, the positions of the 12 cysteines are homologous in the three precursors, meaning that this uniform character of disulfide bridging pattern should lead to similar crystal structures of those three PLA_2_s.

The open-reading frames of the three PLA_2_ proteins were employed in Basic Local Alignment Search Tool (BLAST) searches using the National Center for Biotechnological Information (NCBI) on-line portal. The results indicated that they had a relatively high identity with Group II phospholipase A_2_s from Viperidae snake venoms (Table 1, Figure 2). These studies revealed some very interesting characteristics of the three novel enzymes: PLA_2_-A.C. and PLA_2_-A.S., which both possessed 12 cysteines, showed a disparate alignment map. PLA_2_-A.S. showed some unexpected identities with PLA_2_-A.N. that has 14 cysteines, and they were both obviously most similar to the phospholipases A_2_ classified as Group IIA—the majority cluster. However, PLA_2_-A.C. is nearly identical to phospholipase A_2_ AAR06850.1 from *Bitis gabonica* [21], which falls within the extraordinary Group IIB PLA_2_s that lack one disulfide-bridge conserved in the Group IIA as mentioned in the work of Six *et al.* [2].

### 2.2. Identification and Structural Analysis of PLA_2_ Proteins

**Figure 3:**
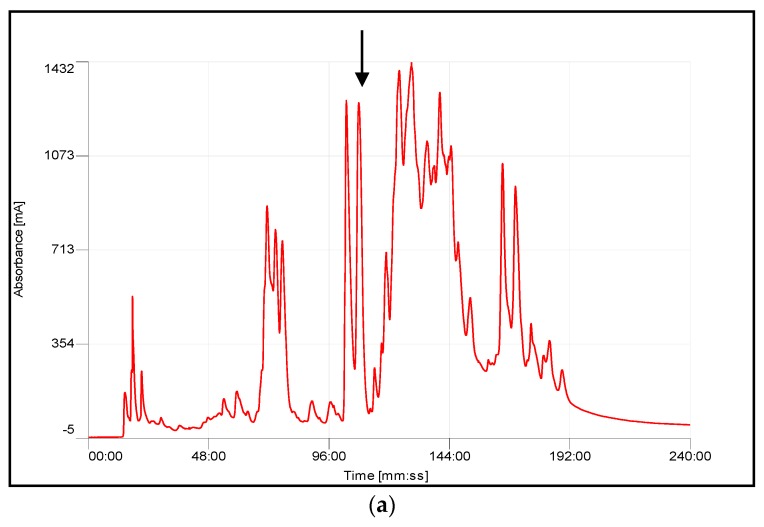

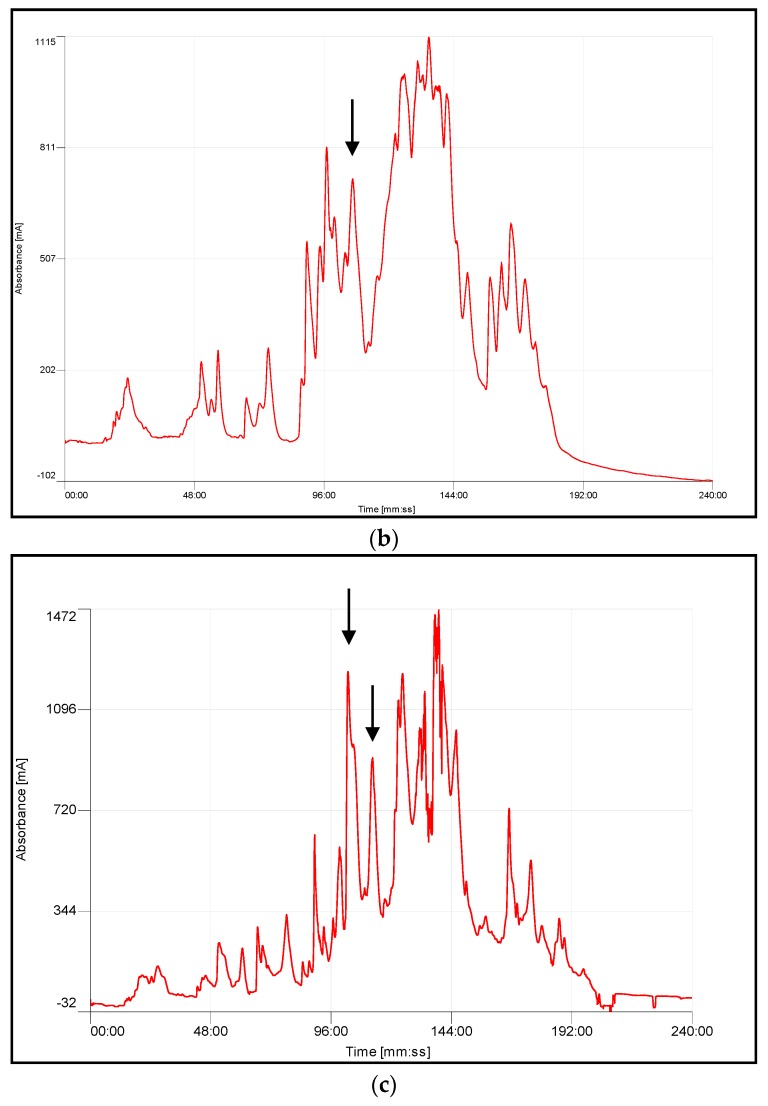
Region of reverse phase High Performance Liquid Chromatography (HPLC) profiles of venoms from (**a**) *A. chlorechis*; (**b**) *A. nitschei*; and (**c**) *A. squamigera*, indicating (arrow) elution position/retention times of PLA_2_-A.C., PLA_2_-A.N. and PLA_2_-A.S., respectively.

A large range of components was successfully resolved following reverse phase High Performance Liquid Chromatography (HPLC) fractionation of the crude lyophilized venoms from *A. chlorechis*, *A. nitschei* and *A. squamigera*. Initial PLA_2_ activity screening of eluted HPLC fractions was performed separately by agarose/egg yolk suspension plates (Supplementary materials Figure S1, Table S1). A number of fractions were identified as possessing phospholipase A_2_ activity and were displayed as isolated peaks in each chromatogram (Figure 3).

The molecular masses of each protein were determined by Matrix-assisted laser desorption/ionization-Time of Flight (MALDI-TOF) mass spectrometry and single major ions with *m*/*z* ratios around 14 KDa were resolved which are in accordance with data from the cDNA-deduced amino acid sequences. The primary structures were subsequently unequivocally confirmed by Liquid chromatography–tandem mass spectrometry (LC/MS/MS) fragmentation sequencing (Table 2).

## 3. Discussion

Whole venoms from *A. chlorechis*, *A. nitschei* and *A. squamigera* were subjected to reverse phase HPLC fractionation, followed by mass spectrometric analysis of each fraction, resulting in consistent identification of PLA_2_ proteins with the predicted masses of mature proteins deduced from cDNA-encoded precursors. It can be seen from Figure 3 that active PLA_2_ fractions from *A. squamigera* venom seems to contain more than one component. However, as shown in Table 2, the spectrum of the peaks at 106 and 113 min differed by only six mass units from the theoretical molecular mass of 13841 Da. The discrepant masses of these two peaks probably could be accounted for by several amino-acid substitutions in the sequences of related isoenzymes which often occur in surface exposed residues which play a key role in recognition of target proteins [22]. Although the spectrum of *A. squamigera* exhibited the probable existence of PLA_2_ enzyme isoforms in the venom, the tiny mass difference of the two peaks indicated a very low possibility. Therefore, we suppose it could just be due to slight differences in hydrophobic properties or the isoelectric points of the two subunits constituting the PLA_2_ homodimer (related to the elutropic theory of reverse-phase chromatography); even external conditions, such as room temperature, stability of ionization, *etc.*, could also be responsible. However, as a consequence of intrinsic molecular diversity, single snake venom can contain up to 16 discrete PLA_2_ homologs with phospholipase activity [23], we cannot affirm that the precursors obtained by molecular cloning in our study represent the only PLA_2_ proteins occurring in these snake venoms.

**Figure 4:**
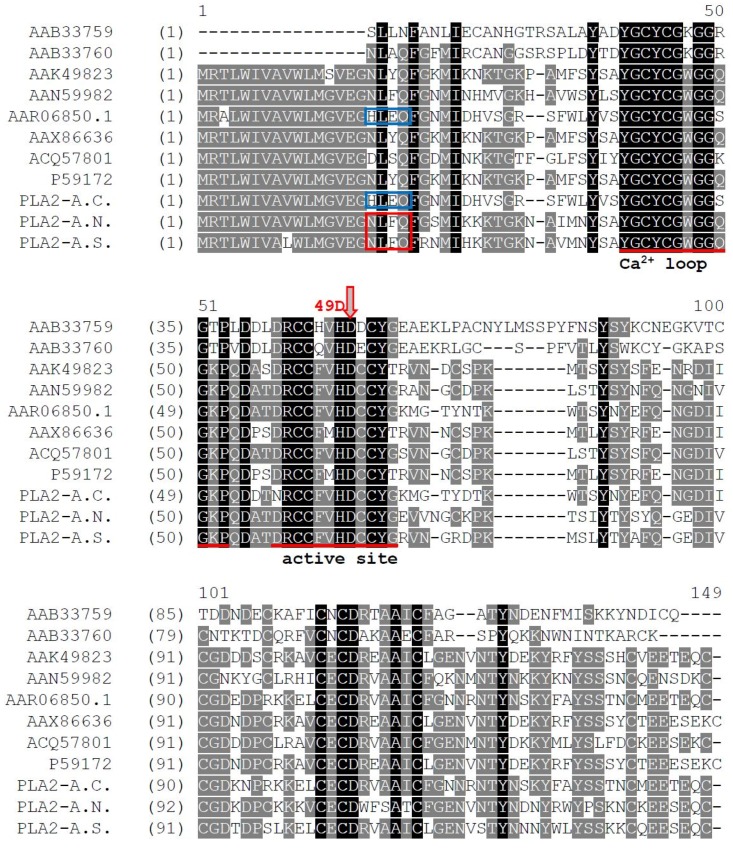
Alignments of PLA_2_-A.C., PLA_2_-A.N., and PLA_2_-A.S. structures and the selected PLA_2_s from the Genbank database. Identical residues are shaded with black and conserved residues with gray. The functional regions are underlined and the highly-conserved active center is exhibited using an arrow. The number of amino acids is illustrated above the first sequence, and the accession numbers of each protein at the National Center for Biotechnology Information (NCBI) Genbank is marked at the front of the array. The *N*-terminal sequences of PLA_2_-A.N. and PLA_2_-A.S. are indicated by a red box; PLA_2_-A.C. and the similar PLA_2_ AAR06850.1 are indicated by a blue box.

Comparative sequence analyses have revealed a high conservation of functionally important domains and disulfide bonding patterns among the different groups of phospholipase A_2_s. This can be seen clearly by our alignment of Groups I and II PLA_2_s from Genbank and PLA_2_-A.C., PLA_2_-A.N. and PLA_2_-A.S., from our original experimental data, as shown in Figure 4. The sequence identity as well as the BLAST data shown in Table 1 proves our hypothesis on the relationship of PLA_2_-A.C., PLA_2_-A.N. and PLA_2_-A.S. PLA_2_-A.N. and PLA_2_-A.S. share the same *N*-terminal sequence NLFQ-, show higher similarity in functional amino-acid sequence and both belong to Group IIA. In contrast, PLA_2_-A.C. has a different *N*-terminal amino acid sequence, HLEQ-, to the previous two PLA_2_s and was identical to AAR 06850.1—the PLA_2_ from Gaboon viper venom [21], which was identified as a Group IIB PLA_2_. Structural comparisons provide a basis for speculation as to the novel phospholipase A_2_ protein metal binding sites, disulfide-bond assignments and residues participating in the catalytic site. These three novel phospholipase A_2_s share, in common with the other PLA_2_s, the Ca^2+^ binding loop, active site, and pivotal amino-acid residues such as the His 48/Asp 49 core and calcium-binding assistants, Tyr 28, Gly 30 and Gly 32 (shown in Figure 4). Many of the similar pre-determinations of former newly-discovered PLA_2_s, derived from such homologies, have been confirmed by enzyme activity assays and by X-ray crystallographic analysis. It is thus justifiable to consider the relationships between primary structures and enzyme properties of these three PLA_2_s and the effects of their key structural frameworks as well as the location and types of residues involved in activity or toxicity.

Little is known about the toxicity of enzymes from venoms of the *Atheris* genus, although the coagulation disturbances, hemorrhaging, acute renal failure and hypertension following snakebite, can all be fatal. Our group has characterized the disintegrin proteins in a previous study [20] and these could explain the platelet aggregation inhibition and blood clotting blocking toxicity of the *Atheris* venoms. It would thus be interesting to predict pharmacological effects and look for the experimental evidence for the involvement of these novel phospholipase A_2_s_._ As direct experimental proof is not yet available, it is not possible to predict the properties of these proteins at the moment, and thus the question remains open.

## 4. Experimental

### 4.1. Materials

Lyophilized venoms from the Western bush viper (*A. chlorechis*), the Great Lakes bush viper (*A. nitschei*) and the Variable bush viper (*A. squamigera*), were obtained from a commercial source (Latoxan, Valence, France).

### 4.2. Molecular Cloning of the Phospholipase A_2_ Precursor-Encoding cDNAs

Five milligrams of each venom sample were separately dissolved in 1 mL of lysis/binding buffer (Dynal Biotech, Wirral, UK); the polyadenylated mRNA was extracted by oligo-dT Dynabeads (Dynal Biotech, Wirral, UK) and was isolated as described by the manufacturer. A SMART^TM^ Rapid Amplification of cDNA ends (RACE) cDNA Amplification kit (BD Clontech, Basingstoke, UK) was then employed. Both 5'-and 3' cDNA ends were synthesized using SMART (Switching Mechanism At 5' end of RNA Transcription) cDNA synthesis technology. The reaction was performed by using a nested universal primer (NUP) and a sense primer (S1: 5'-ATGAGGACTCTCTGGATAGTGGCCG-3') that was designed to be complementary to a highly-conserved domain of the 5'-untranslated region of previously-characterized phospholipase A_2_ cDNAs from related snake species(Accession Nos.: GU012263, AY430405, DQ288157, AM114013 and DQ295886). The DNA fragments of expected size were purified and inserted into the pGEM-T vector system (Promega, Southampton, UK). Plasmid DNAs selected after transformation by *Escherichia coli* (*E. coli*) cells, were amplified and then purified by use of a Rapid HiYield™ Gel/PCR DNA Extraction Kit (RBC Bioscience, Duren, Germany). Sequences of the products were obtained use of an automated 3730 capillary DNA sequencer (Applied Biosystems, Wamington, UK).

### 4.3. Chromatographic Fractionation and Activity Determination

Fractionation of components of all three snake venoms was achieved by gradient reverse-phase HPLC (Cecil CE 4200 Adept, Cambridge, UK), using a Phenomenex C_5_ (300 A, 250 × 10 mm) column. Five milligram samples from each venom were separately reconstituted in 0.05% (*v*/*v*) trifluoroacetic acid (TFA)/water, injected onto the column and eluted with a linear gradient formed from 0.05/99.5 (*v*/*v*) TFA/water to 0.05/19.95/80.0 (*v*/*v*/*v*) TFA/water/acetonitrile, by increasing the percentage of the latter buffer gradually over 240 min at a flow rate of 1 mL/min. The fractions were collected at minute intervals and samples (200 μL) were removed from each fraction in triplicate, concentrated and stored at 4 °C prior to functional studies.

The determination of phospholipase A_2_ (PLA_2_) activity in fractions was achieved by use of agarose/egg yolk suspension plates containing phosphatidylcholine as substrate. A 20% portion of HPLC fractions from each of the snake venoms were concentrated and reconstituted in 0.1% Albumin from bovine serum/phosphate buffer saline (BSA/PBS). The plates were prepared with 4% egg yolk emulsion (Oxoid, Basingstoke, UK) in agarose solution in the presence of calcium, punched with 8 wells per plate and each well was loaded with 10 μL sample. The diameters of cleared egg yolk zones after the 20 h incubation (37 °C) were measured to locate the fractions that possessed PLA_2_ activity.

### 4.4. Identification and Structural Investigations

Reverse phase High Performance Liquid Chromatography (RP-HPLC) fractions possessing PLA_2_ activity were subjected to matrix-assisted laser desorption/ionization, time-of-flight mass spectrometry (MALDI–TOF MS) on a linear time-of-flight Voyager DE mass spectrometer (Perseptive Biosystems, Warrington, UK) in positive detection mode using α-cyano-4-hydroxycinnamic acid as the matrix. Internal mass calibration of the instrument with known standards established the accuracy of mass determination as ±0.1%. Those fractions with masses of approximately 14 kDa coincident with those deduced from cloned cDNAs were digested using trypsin and the resultant tryptic peptides were subjected to primary structural analysis by use of an LCQ Fleet electrospray ion-trap mass spectrometer (Thermo, Cheshire, UK).

### 4.5. Ethical Statement

Venoms used in this study were obtained non-invasively from captive snakes.

## Figures and Tables

**Table 1:** Comparison of the results of similarity searches by use of the Basic Local Alignment Search Tool (BLAST) tool on the Phospholipase A_2_ (PLA_2_) precursor sequences obtained from the venoms of *A. chlorechis* (PLA_2_-A.C.), *A. nitschei* (PLA_2_-A.N.), and *A. squamigera* (PLA_2_-A.S.). The accession numbers shown here are unique identifiers of the recorded protein sequences archived in Genbank.

Protein	Accession Number	Organism	Identities	Group
PLA_2_-A.C.	AAR06850.1	*Bitis gabonica*	96%	GIIB
PLA_2_-A.N.	AAK49823.1	*Echis coloratus*	71%	GIIA
	ACQ57801.1	*Macrovipera lebetina*	71%	GIIA
PLA_2_-A.S.	AAK49822	*Echis coloratus*	73%	GIIA
	ACQ57801.1	*Macrovipera lebetina*	72%	GIIA

**Table 2:** Assignment of the Liquid chromatography–tandem mass spectrometry (LC/MS/MS) identified *Atheris* venom protein fragments (*A. chlorechis*, *A. nitschei* and *A. squamigera*) from fractions shown in Figure 3.

Species	Retention Time (min)	Average Mass Observation	Average Mass Calculation	MS/MS-Derived Sequence
*A. chlorechis*	103–104	13,960.5 Da	13,964 Da	HLEQFGNMIDHVSGR
				CCFVHDCCYGK
				MGTYDTK
				ELCECDR
				VAAICFGNNR
				NTYNSK
*A. nitschei*	106–109	13,975 Da	13,979 Da	NLFQFGSMIK
				NAIMNYSAYGCYCGWGGQGKPQDATDR
				DKDPCK
				VNTYNDNYR
				WYPSK
*A. squamigera*	105–107	13,840 Da	13,841 Da	NLFQFR
				NMIHK
				NAVMNYSAYGCYCGWGGQGKPQDATDR
	113		13,847 Da	NLFQFR CCFVHDCCYGR
				ELCECDR
				CQEESEQC

## Supplementary Materials

The following are available online at www.mdpi.com/2072-6651/8/6/168/s1, Figure S1: Dose response curve of diameter of PLA_2_ clearance zones against log_10_ enzyme concentration of the standard PLA_2_ from honey bee venom. Linear regression analysis was employed, and the regression equation (*Y* = 0.7039*X* − 0.34) was obtained to analyse the equivalent PLA_2_ content of sample venoms. The clearance zones of samples in the agarose/egg yolk suspension plate assay were used to estimate content of PLA_2_s in sample venom fractions. The results are summarised in Table S1. Table S1: Clearance zones and corresponding estimated quantities of PLA_2_ in sample venoms from *A. chlorechis*, *A. nitschei* and *A. squamigera*.
