# Supplementary material for: Molecular Characterization of Three Novel Phospholipase A2 Proteins from the Venom of Atheris chlorechis, Atheris nitschei and Atheris squamigera
Source: Toxins (Basel). 2016 Jun 1;8(6):168. doi: 10.3390/toxins8060168 (PMC4926135; doi:10.3390/toxins8060168)
Supplement: Supplementary file 1 [file toxins-08-00168-s001.pdf]

Supplementary Materials: Molecular Characterization of Three Novel Phospholipase A2 Proteins from the Venom of *Atheris chlorechis, Atheris nitschei* and *Atheris squamigera*

He Wang, Xiaole Chen, Mei Zhou, Lei Wang, Tianbao Chen and Chris Shaw

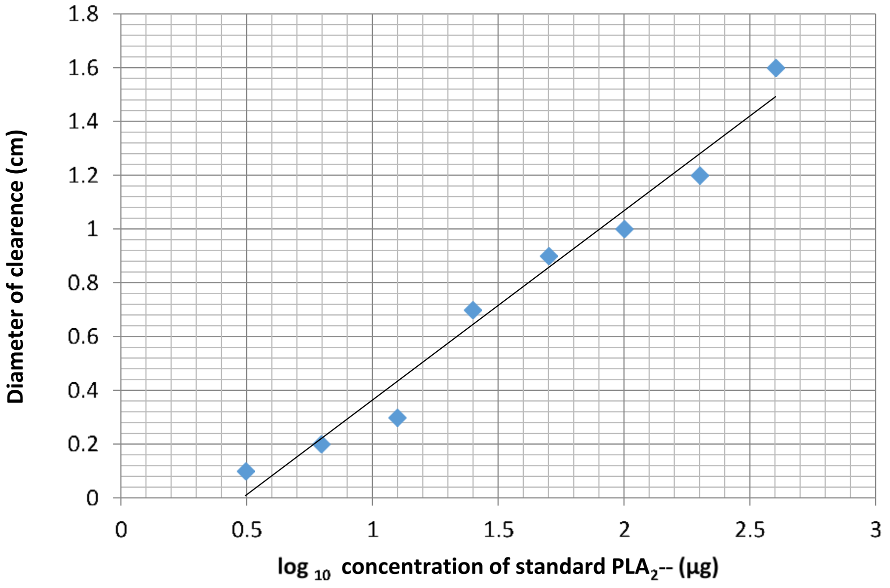

Figure S1. Dose–response curve of diameter of PLA_2 clearance zones against log_{10} enzyme concentration of the standard PLA_2 from honey bee venom. Linear regression analysis was employed, and the regression equation ($Y = 0.7039X - 0.34$) was obtained to analyze the equivalent PLA_2 content of sample venoms. The clearance zones of samples in the agarose/egg yolk suspension plate assay were used to estimate content of PLA_2s in sample venom fractions. The results are summarized in Table S1.

Table S1. Clearance zones and corresponding estimated quantities of PLA_2 in sample venoms from *A. chlorechis*, *A. nitschei* and *A. squamigera*.

Fraction Number		Diameter of PLA_2 Clearance Zone (cm)	Predicted Content of PLA_2 in HPLC Fractions (μg)
A. chlorechis	103	3.25	8.12
	104	3.00	8.71
A. nitschei	106	4.75	14.45
	107	5.00	15.49
	108	5.75	19.95
	109	6.00	21.88
A. squamigera	105	2.50	6.92
	106	3.00	8.12
	107	2.50	6.92
	113	3.25	8.71
